# Synthetic Bax-Anti Bcl_2_ combination module actuated by super artificial hTERT promoter selectively inhibits malignant phenotypes of bladder cancer

**DOI:** 10.1186/s13046-015-0279-6

**Published:** 2016-01-08

**Authors:** Li Liu, Yuchen Liu, Tianbiao Zhang, Hanwei Wu, Muqi Lin, Chaoliang Wang, Yonghao Zhan, Qing Zhou, Baoping Qiao, Xiaojuan Sun, Qiaoxia Zhang, Xiaoqiang Guo, Guoping Zhao, Weixing Zhang, Weiren Huang

**Affiliations:** Key Laboratory of Medical Reprogramming Technology, Shenzhen Second People’s Hospital, The First Affiliated Hospital of Shenzhen University, Shenzhen, China; Urology Department, The First Affiliated Hospital of Zhengzhou University, Zhengzhou, China; Shantou University Medical College, Shantou, China; Department of Urology, Peking University First Hospital, Institute of Urology, Peking University, National Urological Cancer Centre, Beijing, 100034 China; Shanghai-MOST Key Laboratory of Health and Disease Genomics, Chinese National Human Genome Center at Shanghai, Shanghai, China

**Keywords:** hTERT promoter, Bax, Bcl_2_, Synthetic biology, Therapy, Bladder cancer

## Abstract

**Background:**

The synthetic biology technology which enhances the specificity and efficacy of treatment is a novel try in biomedical therapy during recent years. A high frequency of somatic mutations was shown in the human telomerase reverse transcriptase (hTERT) promoter in bladder cancer, indicating that a mutational hTERT promoter might be a tumor-specific element for bladder cancer therapy. In our study, we aimed to construct a synthetic combination module driven by a super artificial hTERT promoter and to investigate its influence on the malignant phenotypes of bladder cancer.

**Methods:**

The dual luciferase assay system was used to verify the driven efficiency and tumor-specificity of the artificial hTERT promoter and to confirm the relationship between ETS-1 and the driven efficiency of the artificial hTERT promoter. CCK-8 assay and MTT assay were used to test the effects of the Bax-Anti Bcl_2_ combination module driven by the artificial hTERT promoter on cell proliferation. Simultaneously, the cell apoptosis was detected by the caspase 3ELISA assay and the flow cytometry analysis after transfection. The results of CCK-8 assay and MTT assay were analyzed by ANOVA. The independent samples *t*-test was used to analyze other data.

**Results:**

We demonstrated that the artificial hTERT promoter had a higher driven efficiency which might be regulated by transcription factor ETS-1 in bladder cancer cells, compared with wild-type hTERT promoter. Meanwhile, the artificial hTERT promoter showed a strong tumor-specific effect. The cell proliferation inhibition and apoptosis induction were observed in artificial hTERT promoter- Bax-Anti Bcl_2_ combination module -transfected bladder cancer 5637 and T24 cells, but not in the module -transfected normal human fibroblasts.

**Conclusion:**

This module offers us a useful synthetic biology platform to inhibit the malignant phenotypes of bladder cancer in a more specific and effective way.

## Background

Bladder cancer is one of the most common types of urologic tumors around the world [1]. Despite methods for early diagnosis of bladder cancer have been emerged, effective treatments are still required [2]. Traditional therapies for bladder cancer are mainly surgery, radiation therapy and chemotherapy, which have dissatisfied side-effect respectively on account of non-specificity and inefficiency [3, 4].

For this reason, it should be obligatory to find a new method for enhancing specificity and availability of bladder cancer therapy.

Synthetic biology is a novel discipline that aims to construct fresh and foreseeable modules, circuits or life entities with the standardized bioparts base on engineering principles [5]. The evolution of synthetic biology provides a favorable platform for the cancer therapy [6].

Therefore, using the theory of synthetic biology to design specific elements is feasible for the cancer treatment. In our previous work [7], the frequency of somatic mutations in the human telomerase reverse transcriptase (hTERT) promoter was about 55.6 % in bladder cancer. The mutant hTERT promoter could activate the expression of hTERT by using the transcription factor v-ets avian erythroblastosis virus E26 oncogene homolog 1 (ETS-1). ETS-1was associated with the progression and angiogenesis of several malignancies [8, 9]. Furthermore, it was reported that ETS-1 expression was high in urothelial carcinomas of the urinary bladder, indicating that it might be a marker of aggressiveness [10]. These discoveries inspired us to design an artificial hTERT promoter as a tumor-specific element regulated by ETS-1.

In the apoptotic pathway, Bax and Bcl_2_ are two important regulator genes [11]. Bcl_2_ regulates cell apoptosis and Bax promotes cell apoptosis [12]. The ratio of Bcl_2_/Bax is closely related to the sensitivity of cells apoptosis. When Bcl_2_ is excessive, cells are protected. On the contrary, when Bax is in excess, cells are susceptible to apoptosis [13]. Moreover, some extracellular or cellular factors were reported to have induced cell apoptosis through reducing Bcl_2_ levels and elevating Bax levels [14–18]. It indicates that the combination of Bax protein and anti-Bcl_2_ molecule can be used to reverse the ratio of Bcl_2_/Bax in bladder cancer.

In this study, we constructed the Bax-Anti Bcl_2_ combination module driven by artificial hTERT promoter which could over express Bax and knockdown of Bcl_2_ and tested the ability of this module in selectively indentifying and killing bladder cancer cells. Our results demonstrated that the Bax-Anti Bcl_2_ combination module driven by artificial hTERT promoter selectively inhibits malignant phenotypes of bladder cancer cells.

## Methods

### Cell lines and cell culture

Human bladder cancer cell lines (T24, 5637, UMUC-3, RT4, J82, SW780) were purchased from the Institute of Cell Biology, Chinese Academy of Sciences (Shanghai, China). Normal human fiber cell (NHF) was kindly provided by Ting Chen, Peking University Shenzhen Hospital, China. T24, UMUC-3, RT4, J82, SW780 and NHF were maintained in DMEM media supplemented with 10 % fetal bovine serum and 1 % antibiotics (100U/ml penicillin and 100 μg/ml streptomycin sulfates). 5637 was maintained in RPMI-1640 media supplemented with 10 % fetal bovine serum and 1 % antibiotics (100U/ml penicillin and 100 μg/ml streptomycin sulfates). All cells were routinely grown at 37 °C in an atmosphere of 5 % CO_2_.

### Design and construction of the artificial hTERT promoter-driven reporter module

Base on the wild-type hTERT promoter sequence, some mutations were introduced to construct the artificial hTERT promoter. The cytosines (C) which were on the upstream −124, −138, −139, and −146 of initiation codon (ATG) were mutated to thymines (T). Furthermore, the adenines (A) which were on the upstream −57 and −189 of ATG were severally mutated to cytosine (C) and guanine (G), and the cytosine (C) which was on the upstream −89 of ATG was mutated to guanine (G). The artificial hTERT promoter has been chemically synthesized. Then the artificial hTERT promoter fragment was cloned into siCHECK^TM^-2 luciferase vector (Promerga, Madison, WI, USA) digested with *BgIII* and *Nhel* to substitute the SV40 promoter. To construct a negative control, the wild-type hTERT promoter fragment was cloned into siCHECK™-2 luciferase vector (Promerga, Madison, WI, USA) digested with *BgIII* and *Nhel* to substitute the SV40 promoter as well. All the detailed sequence information has showed in Table 1.

**Table 1:** Related sequences in the modules

Name	Related sequences
Wild-type hTERT promoter	GGCCCCTCCC TCGGGTTACC CCACAGCCTA GGCCGATTCG ACCTCTCTCC GCTGGGGCCC TCGCTGGCGT CCCTGCACCC TGGGAGCGCG AGCGGCGCGC GGGCGGGGAA GCGCGGCCCA GACCCCCGGG TCCGCCCGGA GCAGCTGCGC TGTCGGGGCC AGGCCGGGCT CCCAGTGGAT TCGCGGGCAC AGACGCCCAG GACCGCGCTC CCCACGTGGC GGAGGGACTG GGGACCCGGG CACCCGTCCT GCCCCTTCAC CTTCCAGCTC CGCCTCCTCC GCGCGGACCC CGCCCCGTCC CGACCCCTCC CGGGTCCCCG GCCCAGCCCC CTCCGGGCCC TCCCAGCCCC TCCCCTTCCT TTCCGCGGCC CCGCCCTCTC CTCGCGGCGC GAGTTTCAGG CAGCGCTGCG TCCTGCTGCG CACGTGGGAA GCCCTGGCCC CGGCCACCCC CGCG
Artificial hTERT promoter	GGCCCCTCCC TCGGGTTACC CCACAGCCTA GGCCGATTCG ACCTCTCTCC GCTGGGGCCC TCGCTGGCGT CCCTGCACCC TGGGAGCGCG AGCGGCGCGC GGGCGGGGAA GCGCGGCCCA GACCCCCGGG TCCGCCCGGA GCAGCTGCGC TGTCGGGGCC AGGCCGGGCT CCCAGTGGAT TCGCGGGCAC AGACGCCCAG GACCGCGCTC CCCACGTGGC GGAGGGACTG GGGACCCGGG CACCCGTCCT GCCCCTTCAC CTTCCGGCTC CGCCTCCTCC GCGCGGACCC CGCCCCGTCC CGACCCCTTC CGGGTTTCCG GCCCAGCCCC TTCCGGGCCC TCCCAGCCCC TCCCCTTCCT TTCCGGGGCC CCGCCCTCTC CTCGCGGCGC GAGTTTCCGG CAGCGCTGCG TCCTGCTGCG CACGTGGGAA GCCCTGGCCC CGGCCACCCC CGCG
Bax	CACCATGGACGGGTC CGGGGAGCAG CCCAGAGGCG GGGGGCCCAC CAGCTCTGAG CAGATCATGA AGACAGGGGC CCTTTTGCTT CAGGGTTTCA TCCAGGATCG AGCAGGGCGA ATGGGGGGGG AGGCACCCGA GCTGGCCCTG GACCCGGTGC CTCAGGATGC GTCCACCAAG AAGCTGAGCG AGTGTCTCAA GCGCATCGGG GACGAACTGG ACAGTAACAT GGAGCTGCAG AGGATGATTG CCGCCGTGGA CACAGACTCC CCCCGAGAGG TCTTTTTCCG AGTGGCAGCT GACATGTTTT CTGACGGCAA CTTCAACTGG GGCCGGGTTG TCGCCCTTTT CTACTTTGCC AGCAAACTGG TGCTCAAGGC CCTGTGCACC AAGGTGCCGG AACTGATCAG AACCATCATG GGCTGGACAT TGGACTTCCT CCGGGAGCGG CTGTTGGGCT GGATCCAAGA CCAGGGTGGT TGGGTGAGAC TCCTCAAGCC TCCTCACCCC CACCACCGCG CCCTCACCAC CGCCCCTGCC CCACCGTCCC TGCCCCCCGC CACTCCTCTG GGACCCTGGG CCTTCTGGAG CAGGTCACAG TGGTGCCCTC TCCCCATCTT CAGATCATCA GATGTGGTCT ATAATGCGTT TTCCTTACGTGTCTGATTCTAGGCGATCG
Anti Bcl_2_ element	AAGGTATATTGCTGTTGACAGTGAGCGCAGGGAGAUAGUGAUGAAGUAATAGTGAAGCCACAGATGTATTACTTCATCACTATCTCCCTTTGCCTACTGCCTCG
Negative control element	AAGGTATATTGCTGTTGACAGTGAGCGCAGCGCAUUCCAGCUUACGUAATAGTGAAGCCACAGATGTATTACGTAAGCTGGAATGCGCTTTGCCTACTGCCTCG

### Construction of ETS-1 shRNA vector

Either the small-hairpin RNA (shRNA) targeting ETS-1 or the negative control shRNA targeting no known sequence were cloned into the pGPU/GFP/Neo vector (GenePharma, Shanghai, China). The sequence of ETS-1 shRNA has been previously provided [19]. The ETS-1 shRNA sequence was 5′-CTGATGTAAGGCAATTAAT-3′.

### Construction of the artificial hTERT promoter- Bax-Anti Bcl_2_ combination module

The cDNA sequence of Bax gene was chemically synthesized and cloned into the artificial-hTERT promoter reporter module digested with *Nhel* and *Xhol* to substitute the Renilla luciferase gene fragment. Meanwhile, Anti Bcl_2_ element, which was constructed by using Bcl_2_ shRNA [20] to replace the mature-miR-30 encoding region of the pre-miR-30 scaffold [21], was inserted into the artificial-hTERT promoter reporter module digested with *Pmel* and *Notl* as well. Eventually, the artificial hTERT promoter - Bax-Anti Bcl_2_ combination module, in which the Bax-Anti Bcl_2_ combination module was driven by artificial hTERT promoter, has been constructed. Similarly, to construct a negative control, the negative control element, which was also constructed by using the sequence of NC shRNA [20] to replace the corresponding region of the pre-miR-30 scaffold, was inserted into the artificial-hTERT promoter reporter module digested with *Pmel* and *Notl*. All the related sequences are shown in Table 1.

### Cell transfection

Cells were plated about 20 h prior to transfection to achieve 80–90 % confluency at the time of transfection. Each device was transfected into cells using LipoFiter™ Liposomal Transfection Reagent (Hanbio, Shanghai, China) according to the manufacturer’s instructions in different experiments.

### Dual luciferase reporter assay

Cells were seeded in 24-well plates (1 × 10^5^/well) and transfected with the relevant designed device. Luciferase activity was detected using the dual luciferase assay system (Promega, Madison, WI, USA) according to the manufacturer’s instructions at 48 h after transfection. In order to calculate the driven efficiency of artificial or wild-type hTERT promoter in bladder cancer cells and NHF, the renilla luciferase activity was normalized to the firefly luciferase activity. The relative luciferase activity (%) of artificial hTERT promoter-driven reporter module co-transfected with ETS-1 shRNA or negative control shRNA was calculated by the following formula: Relative luciferase activity (%) = (luciferase activity of artificial hTERT promoter-driven reporter module co-transfected with ETS-1 shRNA/luciferase activity of artificial hTERT promoter-driven reporter module co-transfected with negative control shRNA) × 100 %. The experiments were performed in duplicate and repeated at least three times.

### CCK-8 assay

The effects of the designed devices on cell proliferation were examined by Cell Counting Kit-8 (Beyotime, Shanghai, China) according to the manufacturer’s instructions. Cells were grown in a 96-well plate (5 × 10^3^/well) for 24 h, transfected with artificial hTERT promoter-Bax-Anti Bcl_2_ combination module or negative control and cultured in normal medium. Zero, 24, 48, or 72 h post-transfection, 15 μl of CCK-8 was added to each well of a 96-well plate and the cells were seed for 1 h. Absorbance was measured at a wavelength of 450 nm using an ELISA microplate reader (Bio-Rad, Hercules, CA, USA). Assays was repeated at least three times.

### MTT assay

The cell proliferation was also examined by using 3-[4, 5-dimethylthiazol-2-yl]-2, 5-diphenyl-tetrazolium bromide (MTT) assay. Cells were seed in a 96-well plate (5 × 10^3^/well) for 24 h, transfected with artificial hTERT promoter-Bax-Anti Bcl_2_ combination module or negative control and cultured in normal medium. At 0, 24, 48, and 72 h after transfection, cells were cultured in 5 mg/ml MTT for 4 h and lysed in dimethyl sulfoxide (DMSO) at room temperature for 10 min. The absorbance in each well was calculated at a wavelength of 490 nm using an ELISA microplate reader (Bio-Rad, Hercules, CA, USA). The experiments were carried out in duplicate and repeated at least three times.

### Caspase 3 ELISA assay

Cells were seeded in 24-well plates (1 × 10^5^/well) and transfected with artificial hTERT promoter-Bax-Anti Bcl_2_ combination module or negative control. Forty-eight hours after transfection, cell apoptosis were detected by calculating the activity of caspase-3 using the Caspase-3 enzyme-linked immunosorbent assay (ELISA) assay kit (Hcusabio, Wuhan, China) according to the manufacturer’s instructions. Absorbance was measured at a wavelength of 450 nm using a microplate reader (Bio-Rad, Hercules, CA, USA). Data were shown as the ratios between the absorbance of artificial hTERT promoter-Bax-Anti Bcl_2_ combination module transfected cells and those of negative control transfected cells. Experiments were performed at least three times.

### Flow cytometry analysis

Cell apoptosis was detected using an Alexa Fluor®488 Annexin V/Dead Cell Apoptosis Kit (Invitrogen, Carlsbad, CA, USA) according to the supplier’s protocols. Cells were seeded in 6-well plates (5 × 10^5^/well). Forty-eight hours post-transfection, cells were harvested, centrifuged, and washed in 1000 μl cold phosphate-buddered saline (PBS) for two times. Then cells in each tube were resuspended in 100 μl 1 × annexin-binding buffer. Five microliter Alexa Fluor® 488 annexin V and 1 μl PI working solution (100 μg/ml) were added to each tube. The tubes were incubated in the dark on ice for 15 min. Cell apoptosis assay was performed immediately on a flow cytometry (EPICS, XL-4, Beckman, CA, USA). Each experiment was done at least three times.

### Statistical analyses

All experimental data from three independent experiments were presented as mean ± standard deviation (SD). All statistical data were analyzed by SPSS 19.0 software (SPSS Inc. Chicago, IL, USA). The data of CCK-8 assay and MTT assay were analyzed by ANOVA. The independent samples *t*-test was used to analyze other data. A P value of less than 0.05 was considered to be statistically significant.

## Results

### Artificial hTERT promoter can drive the expression of downstream gene efficiently and selectively in bladder cancer cells

**Fig. 1:**
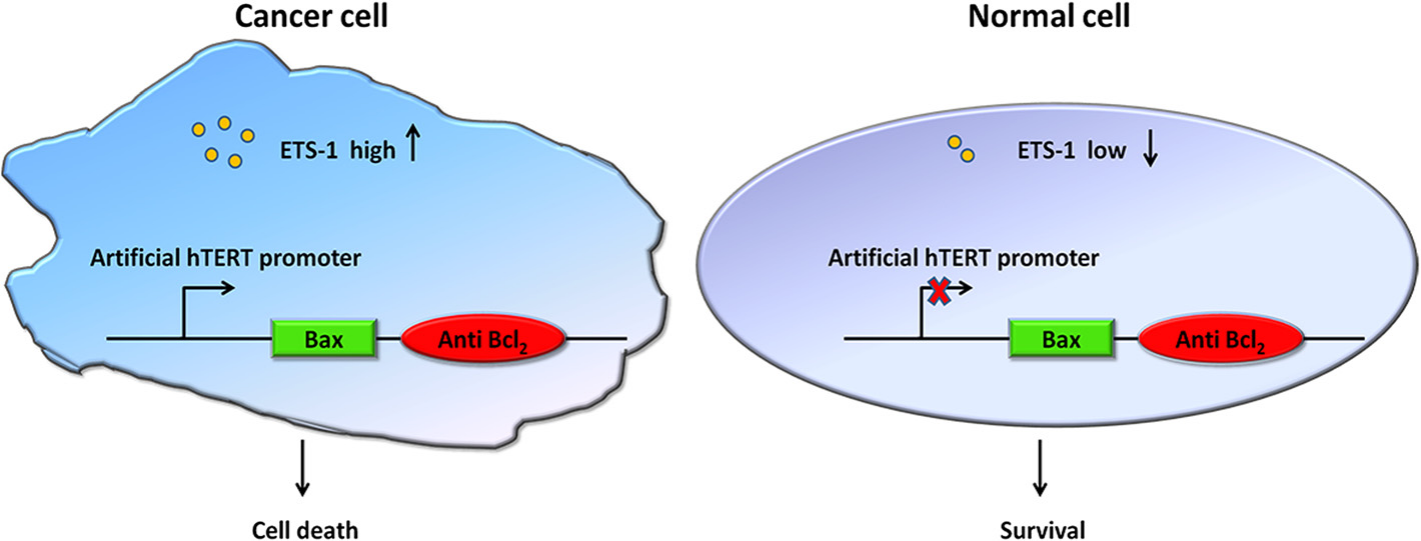
Design and construction of the artificial hTERT promoter-Bax-Anti Bcl_2_ combination module. In the cancer cell, the expression level of transcription factor ETS-1 is high and thus the artificial promoter is active. In contrast, ETS-1 is absent and the artificial promoter is inactive in the normal cells. The apoptotic markers can only be activated in the cancer cells which have a high ETS-1 level

The artificial hTERT promoter was designed and constructed through the approaches of synthetic biology, and the related information was shown in “Methods” section. The designed principle and the action model of synthetic Bax-Anti Bcl_2_ combination module were shown in Fig. 1.

**Fig. 2:**
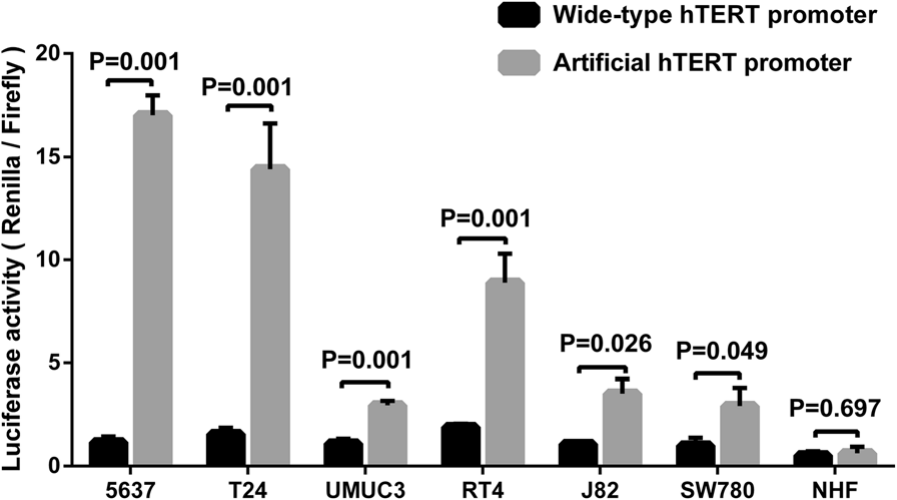
Artificial hTERT promoter can drive downstream gene expression efficiently and selectively in bladder cancer cell. Luciferase activity was detected using dual luciferase assay system at 48 h after transfection. The activity of artificial hTERT promoter was significantly higher than that of wild-type hTERT promoter in bladder cancer cells. Nevertheless, both the activity of artificial hTERT promoter and that of wild-type hTERT promoter were weak in NHF. Data are indicated as mean ± S.D.

Bladder cancer cells and normal human fibroblasts (NHF) were cultured and transfected with artificial hTERT promoter-driven reporter module or wide-type hTERT promoter-driven reporter module. Forty-eight hours later, the activity of the artificial hTERT promoter and wild-type hTERT promoter were detected using dual luciferase assay. As shown in Fig. 2, the driven efficiency of artificial hTERT promoter was significantly higher than that of wild-type hTERT promoter in bladder cancer cells 5637, T24, UMUC-3, RT4, J82 and SW780. The average fold-change was about six-fold. Especially in bladder cancer 5637 cells, the fold-change was up to 14-fold. Oppositely, both the activity of artificial hTERT promoter and that of wild-type hTERT promoter were weak in NHF. There was no difference between the activity of artificial hTERT promoter and that of wild-type hTERT promoter in NHF. The data indicated that artificial hTERT promoter could drive the expression of downstream gene efficiently and selectively in bladder cancer cell.

### Silencing ETS-1 inhibited the activity of artificial hTERT promoter in bladder cancer cells

**Fig. 3:**
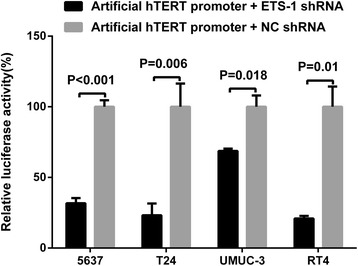
Silencing ETS-1 inhibited the activity of artificial hTERT promoter in bladder cancer cells. Dual luciferase assay system was used to detect the activity of artificial hTERT promoter at 48 h after transfection. The activity of artificial hTERT promoter in the group co-transfected with ETS-1 shRNA was obviously lower than that in the group co-transfected with negative control shRNA. All data are shown as mean ± S.D.

To confirm whether ETS-1 is associated with the driven efficiency of artificial hTERT promoter, bladder cancer cells, 5637, T24, UMUC-3 and RT4, were cultivated and co-transfected with the ETS-1 shRNA and artificial hTERT promoter reporter module or the negative control shRNA and artificial hTERT promoter reporter module. Forty-eight hours post-transfection, dual luciferase assay was used to detect the activity of the artificial hTERT promoter. Compared with the group which was co-transfected with negative control shRNA, the activity of artificial hTERT promoter was respectively decreased by nearly 69 % in bladder cancer 5637 cells, 77 % in bladder cancer T24 cells, 32 % in bladder cancer UMUC-3 cells and 80 % in bladder cancer RT4 cells in the group which was co-transfected with ETS-1 shRNA (Fig. 3). Silencing ETS-1 observably inhibited the activity of artificial hTERT promoter in bladder cancer cell. The data demonstrated that ETS-1 was associated with the driven efficiency of artificial hTERT promoter. ETS-1 is likely to regulate the driven efficiency of artificial hTERT promoter in bladder cancer cells.

### Artificial hTERT promoter-Bax-Anti Bcl_2_ combination module selectively restrained cell proliferation in bladder cancer cells

**Fig. 4:**
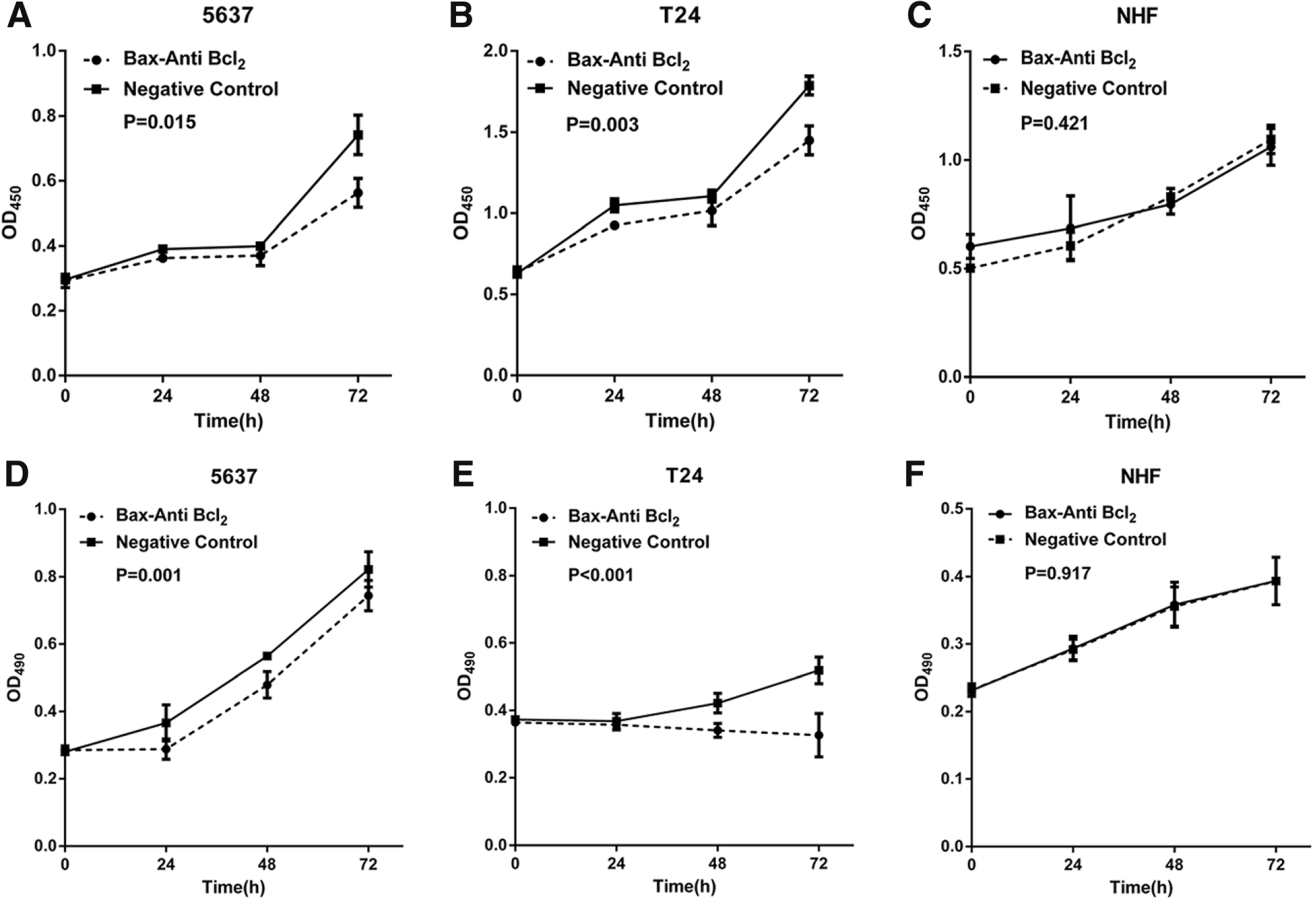
Artificial hTERT promoter-Bax-Anti Bcl_2_ combination module selectively restrained cell proliferation in bladder cancer cells. Cell proliferation assay (CCK-8 and MTT) were performed as described in Methods. The cell proliferation was significantly suppressed in bladder cancer 5637 cells (**a**, **c**) and T24 cells (**b**, **d**) which were transfected with artificial hTERT promoter-Bax-Anti Bcl_2_ combination module (*P* < 0.05), compared with negative control group. However, no significant change was shown in NHF (**c**, **e**) (*P* > 0.05). Data are indicated as mean ± S.D.

Base on the confirmed high efficiency and specificity of artificial hTERT promoter, artificial hTERT promoter-Bax-Anti Bcl_2_ combination module has been constructed to determine whether this module selectively inhibits cell proliferation in bladder cancer. After transfection with artificial hTERT promoter-Bax-Anti Bcl_2_ combination module or negative control, bladder cancer 5637 cells, T24 cells and NHF were analyzed by the cell proliferation assay with CCK-8 and MTT. Compared with the negative control, the cell proliferation was significantly suppressed in bladder cancer 5637 cells (Fig. 4a and d) and T24 cells (Fig. 4b and e) transfected with artificial hTERT promoter-Bax-Anti Bcl_2_ combination module (*P* < 0.05). As expected, there was no difference in the cell proliferation rate between artificial hTERT promoter-Bax-Anti Bcl_2_ combination module and negative control transfected group in NHF (Fig. 4c and f) (*P* > 0.05). These results indicated that artificial hTERT promoter-Bax-Anti Bcl_2_ combination module selectively suppress cell proliferation in bladder cancer cells.

### Artificial hTERT promoter-Bax-Anti Bcl_2_ combination module selectively induced cell apoptosis in bladder cancer cells

**Fig. 5:**
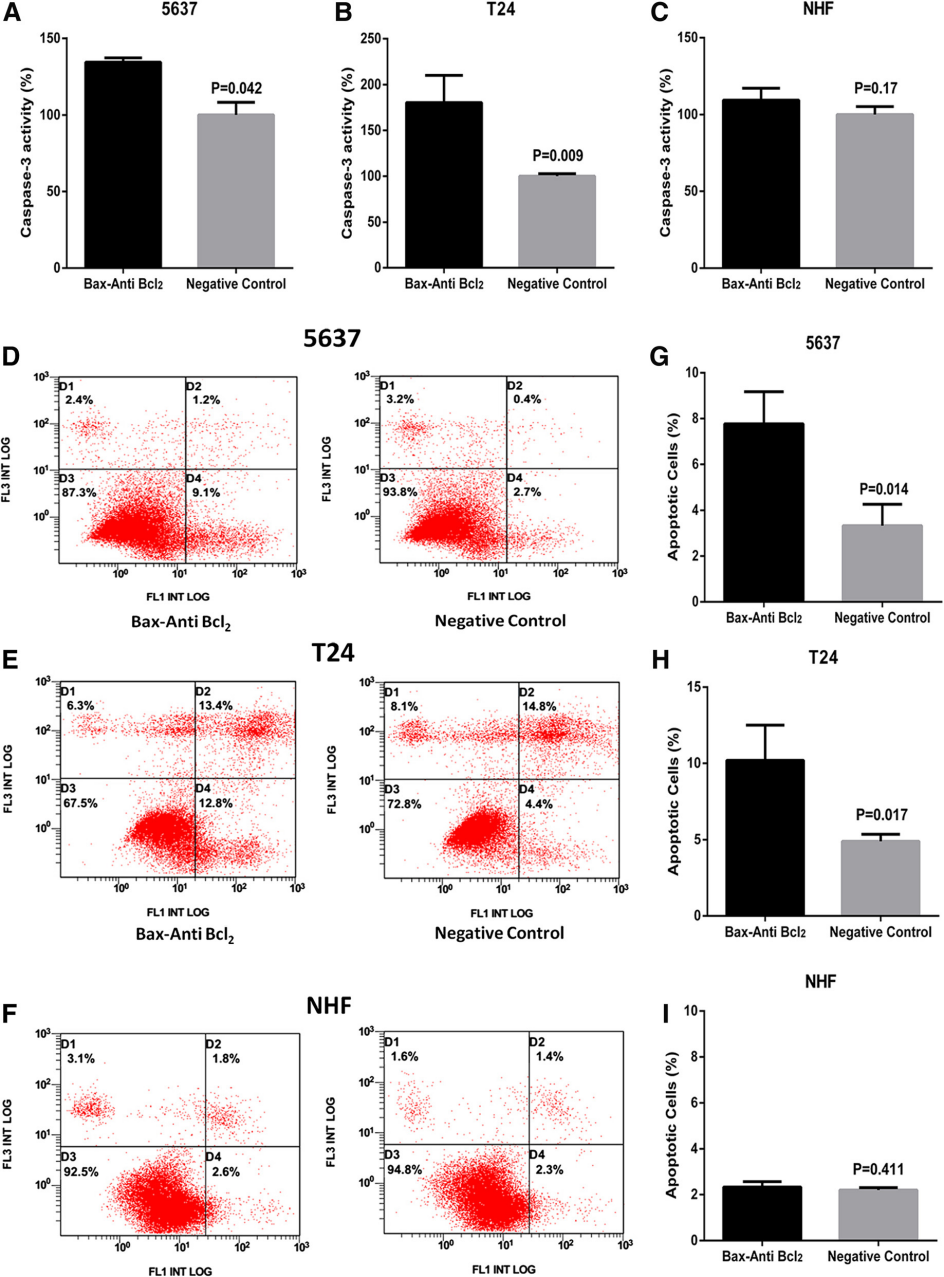
Artificial hTERT promoter-Bax-Anti Bcl_2_ combination module selectively induced cell apoptosis in bladder cancer cells. The cell apoptosis changes were determined by caspase-3 ELISA assay and flow cytometry analysis. The relative activity of caspase-3 was increased in bladder cancer 5637 (**a**) cells and T24 (**b**) cells treated with artificial hTERT promoter-Bax-Anti Bcl_2_ combination module. Cell apoptosis induction were also observed in bladder cancer 5637 (**d**) cells and T24 (**e**) cells treated with artificial hTERT promoter-Bax-Anti Bcl_2_ combination module using flow cytometry analysis. Representative images of flow cytometry analysis in bladder cancer 5637 cells (**g**) and T24 cells (**h**) were also shown. Nevertheless, there was no obvious difference with the relative activity of caspase-3 (**c**) and cell apoptosis induction (**f**, **i**) between artificial hTERT promoter-Bax-Anti Bcl_2_ combination module and negative control in NHF. *D1*, cell debris. *D2*, non-viable apoptotic cell. *D3*, normal cells. *D4*, viable apoptotic cell. All data are presented as mean ± SD

Finally, we explored whether cell apoptosis of bladder cancer cells were selectively induced by artificial hTERT promoter-Bax-Anti Bcl_2_ combination module. At 48 h after transfection of Artificial hTERT promoter-Bax-Anti Bcl_2_ combination module or negative control, the relative activity of caspase-3 and the apoptosis ratio in bladder cancer 5637 cells, T24 cells, and NHF were detected by the caspase-3 enzyme-linked immunosorbent assay (ELISA) assay and the flow cytometry analysis. Induced cell apoptosis was observed in bladder cancer 5637 cells (Fig. 5a, d and g) and T24 cells (Fig. 5b, e and h) transfected with artificial hTERT promoter-Bax-Anti Bcl_2_ combination module. On the contrary, there was no difference in the relative activity of caspase-3 and the cell apoptosis rate between artificial hTERT promoter-Bax-Anti Bcl_2_ combination module and negative control transfected group in NHF (Fig. 5c, f and i). These results confirmed that artificial hTERT promoter-Bax-Anti Bcl_2_ combination module selectively induced cell apoptosis in bladder cancer cells.

## Discussion

Rationally designed synthetic systems that are capable of performing complex functions pave the way for translational applications, including disease diagnostics and targeted therapeutics [22]. With millions of new cases for cancer diagnosis, synthetic biology not only can provide new methods for cancer therapy, but also can study pathways, develop novel diagnostic tools, and address drug delivery issues [6]. Engineered elements for the construction of synthetic biology systems could respond to relative metabolites or other relative cues such as DNA, RNA and proteins [6]. In our team, we devote ourselves to translate the basic cancer research with synthetic biology technology. Usually, we would like to use the promoters, terminators and shRNA/miRNA expression scaffolds as the tools to construct complex devices for cancer treatment [23–25].

Our objective for bladder cancer therapy is to kill cancer cells without affecting the normal cells. So it is necessary to use some tumor- specific genetic parts for the construction of treatment devices. For example, above 85 % of malignancies over-express telomerase, whereas the normal somatic cells doesn’t express telomerase [26]. And various studies also reported that telomerase is up-regulated in about 90 % of bladder cancer cells [27]. The hTERT promoter region is the paramount element for the expression of telomerase [28], in which several factors can regulate the expression of telomerase by occupying the binding motifs of hTERT promoter [29–31]. In addition, the mutations of hTERT promoter were found in almost all kinds of cancers but not in most human normal cells [32, 33]. As the two significant genes in the cell apoptotic pathway, the role of Bax and Bcl_2_ has been well-studied and the ratio of Bcl_2_/Bax was closely related to the sensitivity of cells apoptosis [13]. Some previous researches also reported that several factors could reduce Bcl_2_ levels and elevate Bax levels to induce cell apoptosis [14–18]. Reversing the ratio of Bcl_2_/Bax might affect the bladder cancer cells. All the above information reminds us that we can construct a combination module of Bax over-expression element and Bcl_2_ interference element driven by an artificial hTERT promoter and this module should have a high transcriptional activity and specificity for the bladder cancer treatment.

In this study, to verify the high transcriptional activity and specificity of the artificial hTERT promoter, we constructed the artificial hTERT promoter-driven reporter module at first. The result indicated that artificial hTERT promoter could efficiently and selectively drive the expression of downstream gene in bladder cancer cell. The mutant hTERT promoter can enhance the expression of downstream gene and maintain its tumor-specific feature. The previous research had reported that the 124G > A and 146G > A mutations strongly enhanced the combination between the hTERT promoter and ETS-1 in the mutant T24 cells [7]. Therefore, to investigate whether the activity of the artificial hTERT promoter was also regulated by ETS-1, we co-transfected ETS-1 shRNA and the artificial hTERT promoter-driven reporter module into bladder cancer cells and tested the expression of the dual luciferase. Silencing ETS-1 observably suppressed the activity of artificial hTERT promoter in bladder cancer cell. From our unpublished data, the expression levels of ETS-1 were significantly up-regulated in bladder cancer tissues compared with matched normal tissues. It demonstrated that ETS-1 was associated with the transcriptional activity of artificial hTERT promoter. ETS-1 might regulate the transcriptional activity of artificial hTERT promoter in bladder cancer cells.

Finally, the artificial hTERT promoter-Bax-Anti Bcl_2_ combination module was constructed and tested in the bladder cancer cells and normal human fibroblasts. The results showed that this module could inhibit the cell proliferation and induce the cell apoptosis in bladder cancer cells, but not in the normal human fibroblasts. The Bax-Anti Bcl_2_ combination module driven by artificial hTERT promoter could availably and selectively over-express Bax gene and interference sequence of Bcl_2_ to reverse the ratio of Bcl_2_/Bax for intervention of malignant phenotype of bladder cancer cells. In brief, the Bax-Anti Bcl_2_ combination module driven by artificial hTERT promoter selectively suppresses malignant phenotypes of bladder cancer cells. It would therefore be of great interest to extend this module to clinical research when the technologies for efficient in vivo gene delivery are further developed.

## Conclusions

In summary, the artificial hTERT promoter is a cancer-specific promoter that robustly induces the expression of synthetic modules. The oncogenic signal ETS-1 actuates this artificial cancer-specific promoter. The Bax-Anti Bcl2 combination module can effectively inhibits the malignant phenotypes of bladder cancer.
